# Effectiveness of Toothbrushing Technique for Biofilm Removal and Postoperative Infection Control after Spinal Fusion Surgery: A Retrospective Study

**DOI:** 10.3390/bioengineering10101143

**Published:** 2023-09-28

**Authors:** Sung-ryul Choi, Ji-Won Kwon, Kyung-Soo Suk, Hak-Sun Kim, Seong-Hwan Moon, Si-Young Park, Seung-Eon Moon, Byung-Ho Lee

**Affiliations:** 1 Department of Orthopedic Surgery, Spine and Spinal Cord Institute, Gangnam Severance Hospital, Yonsei University College of Medicine, Seoul 06273, Republic of Korea; srchoi1012@yuhs.ac (S.-r.C.); kwonjjanng@yuhs.ac (J.-W.K.); sks111@yuhs.ac (K.-S.S.); moonse96@yuhs.ac (S.-E.M.); 2 Department of Orthopedic Surgery, Yonsei University College of Medicine, Seoul 03722, Republic of Korea; haksunkim@yuhs.ac (H.-S.K.); shmoon@yuhs.ac (S.-H.M.); drspine90@yuhs.ac (S.-Y.P.)

**Keywords:** spine fusion, postoperative infection, toothbrush, biofilm

## Abstract

This retrospective study was designed to investigate the effectiveness of using a toothbrush, which is commonly used in our daily life, for biofilm removal and infection control in the treatment of spinal infections occurring after spinal fusion surgery. Currently, a biofilm is thought to form on the surface of the metal inserted during spine fusion surgery. We aim to determine the differences in clinical outcomes between using and not using a toothbrush to remove biofilm while performing conventional drainage, curettage, and debridement. A total of 1081 patients who underwent anterior or posterior spinal fusion surgery between November 2018 and October 2022 were screened. The study included 60 patients who developed surgical site infection and underwent incision and drainage surgery either with a toothbrush (*n* = 20) or without a toothbrush (*n* = 40). Failure of infection control that requires revision surgery occurred in 2 patients (10%) in the Toothbrush group and in 14 patients (35%) in the No-Toothbrush group (*p* = 0.039). Thus, the rate of additional surgery was significantly lower in the Toothbrush group. Additionally, normalization of c-reactive protein levels occurred significantly faster in the Toothbrush group (*p* = 0.044). Therefore, using a toothbrush to treat spinal infections following spinal fusion surgery appears to have beneficial mechanical debridement effects, resulting in improved clinical results, which were also confirmed based on the electron microscopic images.

## 1. Introduction

Surgical site infections (SSIs) are a common healthcare-associated infection and constitute up to 10–15% of operations, resulting in considerable strain on healthcare resources [1]. SSIs are the most common type of nosocomial infection, which are infections that occur when a patient’s immune system is unable to protect against resident bacteria in healthcare institutions [2]. The reported incidence of SSI after spinal surgery varies considerably, from 0% to 15%. SSIs are, thus, a relatively common and potentially catastrophic complication following spinal surgery, leading to increased morbidity, mortality, and healthcare costs [3,4]. Additionally, surgical site infection after spine surgery is becoming a leading cause of re-admission [5].

Spinal surgery is challenging and tends to be invasive, resulting in a higher risk of infection than with other orthopedic operations. Fusion surgery is associated with a higher infection rate than other spinal surgeries, such as simple discectomy and posterior laminectomy. Among spinal fusion operations, the highest infection rate is seen with anterior–posterior combined spinal fusion [6,7,8].

Treatment of spinal infections requires intravenous antibiotics, and additional surgical intervention may also be necessary. Traditional treatment protocols for spinal SSI involve early recognition, debridement, irrigation, and administration of organism-specific antibiotics [9,10,11,12]. One of the main causes of treatment failure in spinal infections is biofilm formation [13]. These biofilms, which can form on vertebrae, other spinal structures, or implants, contribute to the chronicity and treatment resistance of spinal infections [14,15,16]. Additionally, biofilm can cause pseudoarthrosis and persistent back pain, even if the wound is sterile [13].

There is a similarity between removing dental plaque with a toothbrush in our daily life and attempting to remove biofilm for treatment of infection [17]. In terms of oral hygiene, both mouthwashes and toothbrushes play important roles. Toothbrushes are especially effective for removing foreign substances, as well as plaque deposits adhering to the teeth, which are similar to biofilms occurring in spinal infections [18,19,20].

Mechanical debridement using a toothbrush, which is commonly performed in our daily life, has not been previously attempted for the treatment of spinal infections. Therefore, we applied this approach for biofilm removal, based on the similarity between dental plaques and biofilms in spinal infections. This retrospective clinical research series describes our novel surgical treatment technique and its effectiveness as a therapeutic option for postoperative spinal infection.

## 2. Materials and Methods

### 2.1. Patient Selection

This study included adults who developed a postoperative SSI after anterior or posterior spinal fusion for degenerative disease of the cervical, thoracic, or lumbar spine at the Severance Hospital or Gangnam Severance Hospital between November 2018 and October 2022. We included patients with a postoperative spinal infection who failed conservative treatment (e.g., intravenous and/or oral antibiotics, bed rest, analgesics, bracing) and required surgical treatment because of the possibility proceeding of a septic condition, such as sepsis, spinal cord, or nerve root compression with progressive neurological deficit. Conservative treatment failure in postoperative spinal infections is indicated by exacerbation of pain, progression of neurologic impairment, elevated c-reactive protein (CRP), and bony changes in radiologic images [10].

Patients with an infectious disease (e.g., infectious spondylitis, epidural abscess) as the indication for their initial fusion surgery, individuals with primary or metastatic cancer, and those receiving immunosuppressants or antibiotics before hospitalization were excluded from the study. Furthermore, patients with potential instabilities, such as vertebral body collapse or mechanical failure due to screw loosening, inherently requiring reconstructive revision surgery at the time of incision and drainage (I&D) surgery, thus, have been excluded. Of the 1081 patients who underwent anterior or spinal fusion during the study period, 60 patients developed a postoperative spinal infection and met the inclusion criteria for this study.

The study was conducted in accordance with the guidelines of the Declaration of Helsinki and approved by the Institutional Review Board of Gangnam Severance Hospital, College of Medicine, Yonsei University (IRB No. 3-2022-0368, 14 November 2022).

### 2.2. Preparation and Patient Treatment

The toothbrush used in this study was WANGTA, manufactured by SamJung Corporation (Seoul, South Korea). This popular product was purchased at a nearby market and was brought into the operating room area without opening the packaging and then subjected to ethylene oxide gas sterilization for 15 h before use. Any other commercially available toothbrush can be also used after sterilization in the operating room. This sterilization was performed using the EOG 500 sterilizer (Delta Medical) at a pressure of 15 PSI and a temperature of 55 °C. The toothbrush was immediately disposed of after the surgery.

The 60 patients with SSI after spinal fusion surgery presented with various clinical manifestations, in combination with deep soft-tissue infection, such as epidural abscess, pseudoarthrosis, infectious spondylodiscitis, and biofilm formation. Our research and treatment focused on deep infections, based on the premise that these infections created sufficient biofilm around the implants to result in resistance to conservative treatment alone [21,22].

When clinical symptoms of infection appeared, culture samples were obtained via computed tomography (CT)-guided biopsy or ultrasonography-guided aspiration, and intravenous treatment with a first-generation cephalosporin antibiotic started empirically before the etiologic organism was identified. After the organism and sensitivity results were available, specific antibiotics were initiated through consultation with our institution’s Infectious Disease Department. Patients in serious condition were treated with more aggressive empirical antibiotic regimens (even before any culture results were obtained), such as dual-agent therapy with a third-generation cephalosporin or fluoroquinolone, plus clindamycin or vancomycin [23].

**Figure 1:**
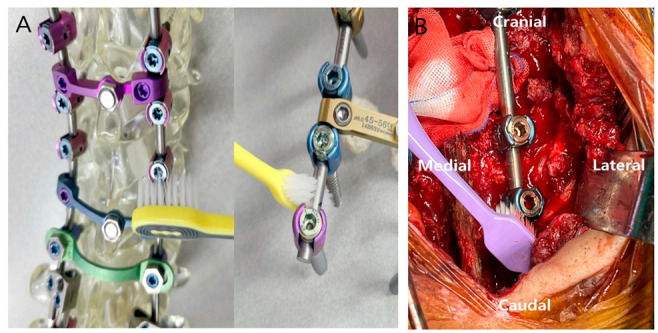
Diagrams of mechanical debridement using a toothbrush for post-spinal fusion infection (**A**). Intraoperative photograph showing removal of biofilm from implants using a toothbrush (**B**).

**Figure 2:**
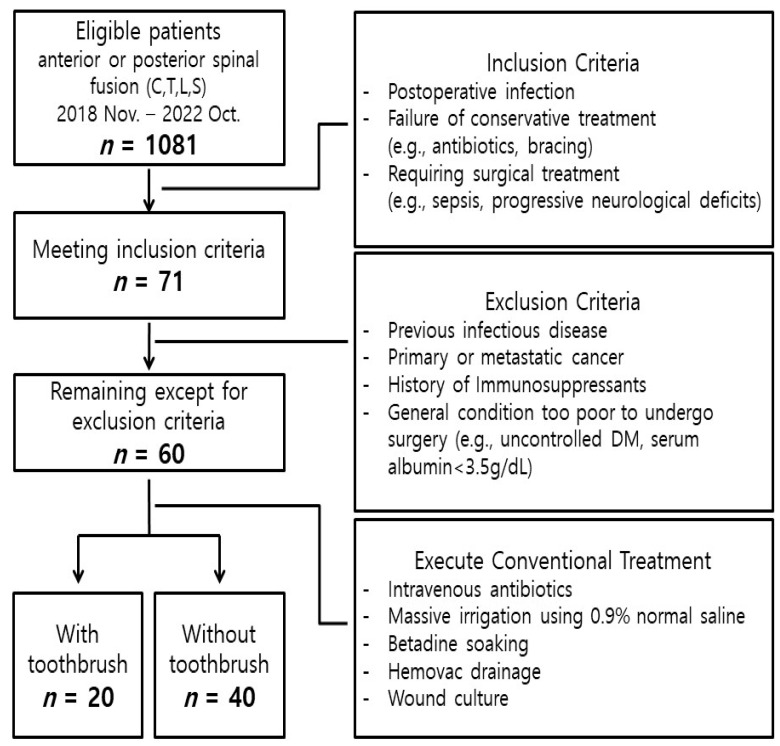
Flowchart of patients included and excluded in this study. Abbreviations: C, cervical; T, thoracic; L, lumbar; S, sacral; Nov, November; Oct, October; DM, diabetes mellitus.

All patients underwent conventional I&D surgery, including irrigation with massive volumes of 0.9% normal saline, betadine soaking, placing a drainage catheter, and taking cultures in the wound. Samples for wound culture were obtained with a swab inserted as deep as possible into the wound. In 20 patients (Toothbrush group), necrotic tissue was debrided, and biofilm was removed with a toothbrush around the inserted implant (including rod and screws) before the last irrigation and wound closure. The model used during the surgery is shown in Figure 1. The control (No-Toothbrush) group of 40 patients underwent only the aforementioned conventional treatment, without using a toothbrush. The overall study flowchart is shown in Figure 2.

### 2.3. Patient Evaluation

A number of patient characteristics were collected, including sex, age, height, weight, body mass index, history of smoking, presence of comorbidities (hypertension, diabetes, kidney disease, respiratory disease), number of instrumented fusion level(s), estimated blood loss, red blood cell (RBC) transfusion during initial fusion surgery, and white blood cell (WBC) counts and absolute neutrophil counts (ANCs) before first I&D surgery. We also collected data regarding the time from fusion surgery to SSI diagnosis, from SSI diagnosis to first I&D surgery, serum glucose and albumin after I&D surgery [24], and time to drainage catheter removal and volume of drainage after I&D surgery. We also recorded the pathogenic microorganisms detected from wound cultures obtained during I&D surgery, as well as the antibiotics that were maintained throughout hospital stay, including the duration of the intravenous antibiotics perioperative period of initial I&D surgery (which were typically continued until normalization of c-reactive protein levels). Patients were followed for evidence of infection by evaluating physical signs at the surgical site, body temperature, and levels of hematologic markers (WBC and ANC counts, erythrocyte sedimentation rate (ESR), and CRP). After discharge, to ensure the stable controlled status of the infection and keep the total duration of antibiotics usage for 6 weeks, oral antibiotics were prescribed for about 2 weeks [10].

The success of SSI treatment was assessed by observing normalization of laboratory test results (e.g., WBC and ANC counts, inflammatory markers) and improvement in vital signs (e.g., body temperature). Treatment failure was defined as the need for additional surgery, such as screw removal or insertion, or extension of the fusion to upper or lower levels requiring an additional reconstructive bone graft [25,26].

### 2.4. Statistical Analysis

We conducted a normality test (Shapiro–Wilk test) for continuous variables and found that most variables did not meet the assumption of normality (except age). Therefore, we opted for non-parametric statistical methods because of violation of the normality assumption for most variables.

For continuous variables, we presented the data as median, minimum, and maximum values and used the Mann–Whitney U test to investigate differences between the Toothbrush group and the No-Toothbrush group. For categorical variables, we reported the data as frequencies and proportions and used the chi-square test (or Fisher’s exact test, if applicable) to investigate differences between these two groups. Statistical analysis was performed using SAS version 9.4 (SAS Institute, Cary, NC, USA). Statistical significance was set at a *p*-value < 0.05.

## 3. Results

### 3.1. Patient Characteristics

Patient characteristics, including descriptive statistics of both patient groups, are summarized in Table 1. A total of 36 males and 24 females were included in the study, with a median age of 69 years (range, 55–89 years). The overall rates of hypertension, diabetes mellitus, kidney disease, and respiratory disease were 55% (*n* = 33), 46.67% (*n* = 28), 26.67% (*n* = 16), and 15% (*n* = 9), respectively. The site of surgery was cervical vertebrae in 7 patients (11.67%), thoracolumbar vertebrae in 22 patients (36.67%), lumbar vertebrae in 20 patients (33.33%), and lumbosacral vertebrae in 11 patients (18.33%). Before initial I&D surgery, the median preoperative WBC count was 7.12 × 10^3^ cells/µL (range, 2.78–18.62 × 10^3^ cells/µL), and the median preoperative absolute neutrophil count (ANC) was 4217.80 cells/µL (range, 1278.49–7944.78 cells/µL). The median time from fusion surgery to SSI was 75 days (range, 10–500 days), and the median time from SSI diagnosis to the first I&D surgery was 8 days (range, 1–90 days). Age was the only baseline characteristic that differed significantly between the Toothbrush and No-Toothbrush groups.

### 3.2. Clinical Outcomes

There was one death and one loss to follow-up in the 60 patients included in this study. *Staphylococcus aureus* and *Staphylococcus epidermidis* were the bacteria most frequently identified in culture samples obtained during initial I&D surgery. Ceftriaxone and vancomycin were used empirically in three and five patients, respectively, with identified bacteria in a nasal swab conducted by other hospitals just before visiting our hospital. The bacteria detected during initial I&D surgery of each patient group are shown in Table 2.

Among all patients, the median time of drainage catheter removal after I&D surgery was 7 days (range, 5–14 days), the median time to CRP normalization after I&D surgery was 24 days (range, 10–90 days), and the median duration of antibiotic use in the hospital perioperative initial I&D surgery was 25 days (range, 10–90 days). The median time from fusion surgery to diagnosis of SSIs was 75 days (range, 10–500 days), and there was no significant statistical association (*p* = 0.0796) between the Toothbrush group and the No-Toothbrush group. These clinical outcomes are summarized in Table 3.

Only 2 of the 20 patients (10%) in the Toothbrush group were considered treatment failures and required revision surgery including screws change; fusion extension to upper or lower vertebral segments; and additional bone graft, irrigation, and drainage surgery for infection control. However, 14 of the 40 patients (35%) in the No-Toothbrush group required revision surgery, and it showed a statistically significant difference. In addition, the Toothbrush group had a significantly shorter time to CRP normalization than the No-Toothbrush group (*p* = 0.044).

Of note, patients who had instability due to implant loosening were excluded from this study because revision surgery was almost routinely performed in these patients, and one of the goals of our treatment was to prevent progression to additional screw loosening and change because of biofilm formation. The following is a brief overview of two representative cases, in which we did or did not use a toothbrush based on this treatment policy.

**Figure 3:**
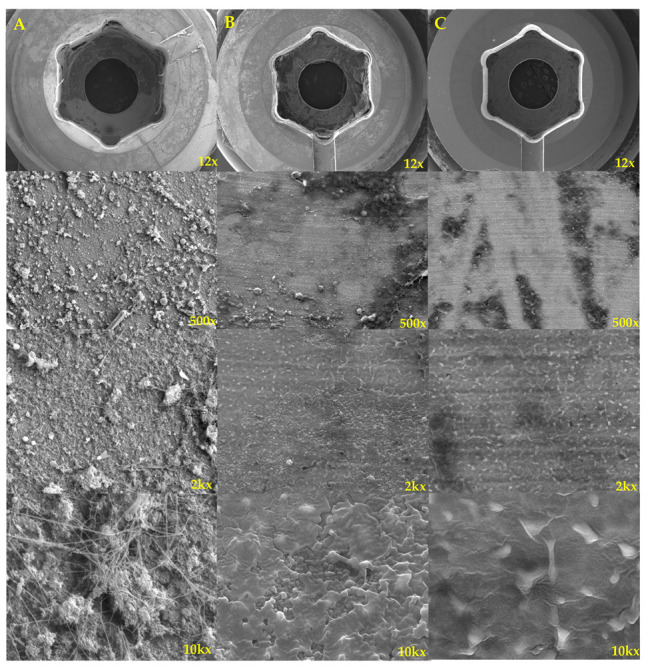
This figure was enlarged using an electron microscope at 12, 500, 2k, and 10k magnifications of the pedicle screw cap from postoperative infected patient in experimental condition. (**A**) Electron micrograph of the screw cap observed before brushing; biofilms are observed. (**B**) Electron microscope of the screw cap irrigated with normal saline after soaking in betadine for 30 min without brushing [27]. Shrunk cell debris and biofilm still remain. (**C**) Electron micrograph of the screw cap after brushing more than 120 s. Most cell debris and biofilm are removed, and a clean cap surface is observed.

The electron micrograph presented in Figure 3 illustrates the condition of surgical instruments used on a postoperative patient with an infection. This depiction encompasses three distinct states: the instruments’ condition prior to brushing, their state following a brushing duration exceeding 120 s, and their appearance after undergoing irrigation with normal saline following a 30 min betadine soak, all under controlled experimental conditions [27].

Many biofilms and biologic debris were observed in the electron microscope before brushing, but most of the biofilms and debris disappeared in the electron microscope after brushing. In the case of electron microscopy after betadine soaking, cells and debris were shrunk, but dead cell debris and biofilms remained enough to work as a scaffold for secondary biofilm formation and regrowth of surviving bacteria.

#### 3.2.1. Toothbrush Group Patient: Successful Treatment

**Figure 4:**
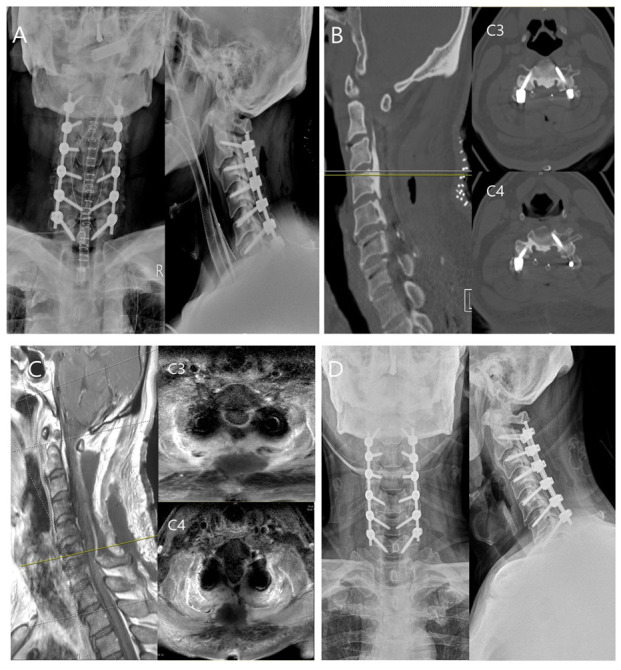
A 63-year-old male presented with reduced sensation below T4 and bilateral hand clumsiness after falling down stairs. Preoperative computed tomography showed K-line (-), C2–C7 mixed-type ossification of the posterior longitudinal ligament, with spinal canal encroachment. Urgent surgical treatment (decompressive laminectomy and posterior fusion with pedicle screws at C2–C7) was performed, and the immediate postoperative X-ray is shown in (**A**). However, the patient developed a continuous high fever and significant CRP elevation on postoperative day 4; CT images showed no bony destruction, but swelling and gas formation of soft tissue (**B**). Magnetic resonance T1 images showed a fluid collection around the deep soft tissue, consistent with an acute postoperative infection (**C**). I&D surgery using a toothbrush was performed 10 days after fusion surgery. This resulted in infection control and symptom improvement. The patient was discharged 1 month after the I&D surgery. One-year postoperative X-rays showed successful infection control, good alignment, and bony fusion (**D**). The patient provided informed consent to publish all of his clinical images.

A 63-year-old male presented to the emergency room with reduced sensation below T4 and bilateral hand clumsiness after falling down stairs. He was diagnosed with C2–C7 ossification of the posterior longitudinal ligament on X-ray and CT. Urgent surgical treatment (decompressive laminectomy and posterior fusion with pedicle screws at C2–C7) was performed. On postoperative day 4, he developed a continuous high fever (approximately 39 °C) and significant CRP elevation (230.2 mg/L [normal, 0–8 mg/L]). To evaluate the etiology of his fever, blood cultures and cervical magnetic resonance imaging (MRI) were performed. The MRI revealed a fluid collection at the surgical site, consistent with an SSI. Analysis of fluid obtained by ultrasound-guided aspiration revealed an elevated WBC, low glucose <10 mg/dL, and Gram-positive cocci. Cefazolin was begun as empirical treatment. I&D using a toothbrush was performed 10 days after the fusion surgery. Symptoms improved, and the CRP decreased to the normal range (1.6 mg/L) within 3 weeks of the initial I&D surgery. One month after the initial I&D surgery, the patient was discharged to home with oral antibiotics, and at the 6-month follow-up in the outpatient department, he had no specific symptoms or radiologic findings of infection, such as implant loosening (Figure 4).

#### 3.2.2. No-Toothbrush Group Patient: Treatment Failure

**Figure 5:**
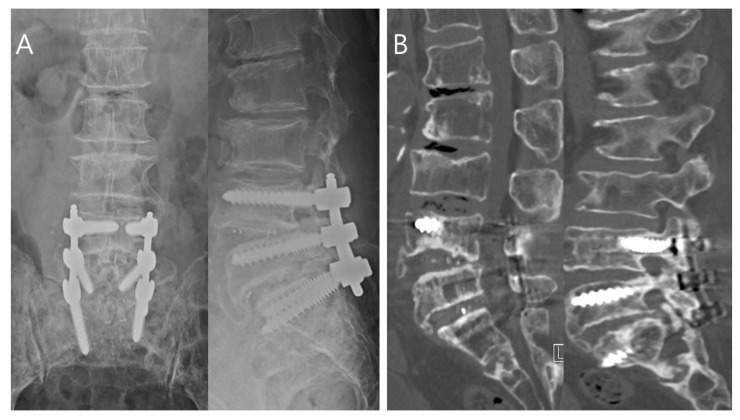

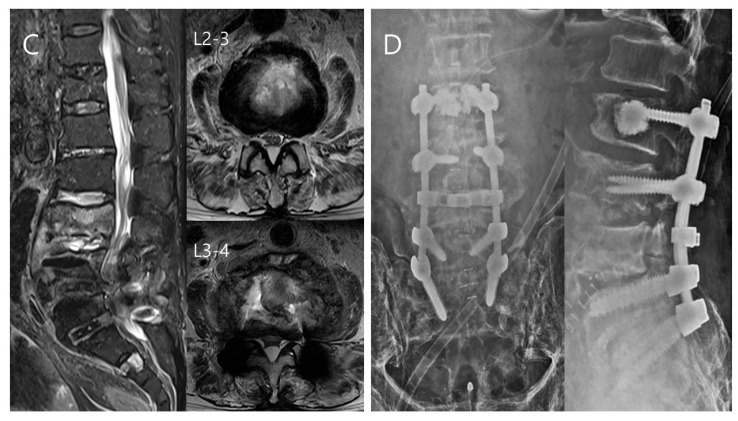
A 70-year-old female presented with worsening low back pain and radiating pain from the left buttock to the posterior thigh. She was diagnosed with L4–L5–S1 spinal stenosis and underwent partial laminectomy, posterior lumbar interbody fusion, and posterior instrumentation at L4–L5–S1. Four months after surgery, she presented to the hospital with sudden onset of severe back pain, and examination of the surgical site revealed swelling, fluctuance, and warmth. We suspected a late postoperative infection, which was confirmed by X-ray (**A**) and computed tomography (**B**). There was no implant loosening finding, such as osteolysis around screws on the radiologic evaluations; she underwent first incision and drainage surgery a week after the SSI diagnosis without a toothbrush, which resulted in temporary improvement. However, during the next 2 months, she was noted to have persistent signs of postoperative infection, and imaging studies showed spondylodiscitis, destruction of the upper vertebra endplate, and progression of the kyphotic deformity (**C**). She eventually underwent revision surgery with screw removal and additional instrumentation for extended fusion to L2; her postoperative X-rays are shown in (**D**). The patient provided informed consent to publish all of her clinical images.

A 70-year-old female presented to the outpatient department with worsening low back pain (visual analogue scale [VAS] score > 6/10) and pain radiating from the left buttock to the posterior thigh. She was diagnosed with L4–L5–S1 spinal stenosis and underwent partial laminectomy, posterior lumbar interbody fusion, and posterior instrumentation at L4–L5–S1. Her radiating pain improved immediately after fusion surgery. The patient was later discharged from the hospital, and no specific findings were observed during early outpatient follow-up. Four months after fusion surgery, she returned to the hospital with sudden-onset back pain (VAS score > 8/10). Physical examination revealed a body temperature of 38.8 °C, as well as swelling, fluctuation, and warmth at the surgical site. CRP was 98.12 mg/L (normal, 0–8 mg/L) after 4 months. The patient was diagnosed with late SSI, and conservative treatment was begun. There was no improvement in symptoms after a week; only traditional I&D surgery was performed, with no toothbrush. Symptoms resolved temporarily, but at 2-month follow-up, she was noted to have continued findings of infection, as well as kyphotic deformity progression of the upper vertebral segment. Revision surgery was eventually performed, with screw removal and additional instrumentation for bone grafting with fusion extension to L2 (Figure 5).

## 4. Discussion

Currently, the proportion of people aged 65 years or older in Korea is approximately 18% of the total population, and our country is transitioning from an aging society to a superaged society. There is a 90% probability that life expectancy at birth among South Korean women in 2030 will be more than 86.7 years (the same as the highest worldwide life expectancy in 2030) and a 57% probability that it will be more than 90 years [28]. Consequently, an increased prevalence of spinal degenerative changes, including loss of disc height, ligamentum flavum hypertrophy, and facet joint arthritis (e.g., spinal stenosis), is expected to increase the demand for spinal fusion surgery, and postoperative infections can be a significant and challenging problem, particularly in the elderly population with multiple comorbidities [29,30,31,32].

Typical treatment for postoperative spinal infections consists of long-term antibiotics, but this can lead to adverse effects, including the emergence of antibiotic-resistant strains, nephrotoxicity, hepatotoxicity, injection site inflammation, allergies, and pseudomembranous colitis [10]. Vancomycin powder has been applied at the surgical site during spinal surgery to prevent postoperative spinal infections, but complications related to vancomycin can also occur, and there is ongoing research into alternative antibiotics, such as Taurolidine [4]. However, the longer antibiotics are used for spinal infections and the more overlap between antibiotics, the greater the morbidity and mortality [33]. Therefore, it is necessary to develop easy and simple methods for rapid biofilm removal.

Despite preventive efforts, spinal fusion surgery may still result in spinal infection in a certain percentage of patients, which may require further procedures (such as revision surgery and removal of spinal fixation devices, as well as the insertion of new devices) if conservative treatment with antibiotics is not effective [34,35]. If infection cannot be effectively controlled even with revision surgery, multiple extensive and repeated operations may be required, resulting in decreased patient quality of life, increased financial burden for the family, and even catastrophic outcomes, such as patient death [36,37]. These issues also increase societal costs.

It is also important to note that treatment algorithms differ between acute and chronic spinal infections. In this study, retention of the implant was possible during the acute phase and depended on the degree of solid bone fusion during the chronic phase [23]. Even early SSIs can cause serious morbidity after anterior cervical surgery, as they can lead to major complications, such as esophageal injury, making multiple debridement necessary [38].

Biofilms are slimy matrices consisting of a complex community of microorganisms (mostly bacteria, but also potentially fungi), as well as extracellular polysaccharides, DNA fragments, and lipoproteins. These biofilms adhere to surfaces and provide a protective matrix of polymeric substances [39]. Their complex structure can limit the penetration of antimicrobials into the biofilm and may lead to the emergence of resistance mechanisms [40]. To address this, high-dose antibiotic therapy, up to 500–5000 times that required to treat planktonic bacterial infections, may be necessary [41,42]. With spinal fusion surgery, biofilms can form on the surface of implanted devices. Thus, biofilms increase the likelihood of unsuccessful conservative antibiotic treatment of SSIs after spinal fusion surgery.

Until now, research methods to address the issue of biofilms in spinal surgery have included techniques such as sonification and reinsertion of screws after soaking in povidone for more than 30 min. According to Bumpass et al. [43], a 30 min povidone–iodine soak was used to treat contaminated pedicle screws, and the rate of bacterial decontamination was 98%; but, however, there is complex multiaxial screw geometry when a biofilm-forming, resistant MRSA bacterium is present. According to Hersh et al. [44], most surgeons prefer maintaining the instrument when the risk of spinal instability is judged to be high in the early infection phase. However, as in this study, if toothbrushing is performed in the early infection phase, infection can be well controlled without instrument removal. Therefore, there is a need for technology that can remove biofilms more easily and efficiently, and it has been recognized that there is a similarity between the dental plaques and biofilms that form on orthopedic implants. We, therefore, proposed the use of a toothbrushing technique to remove biofilms [45,46]. We found that surgery to remove biofilm through mechanical debridement with a toothbrush after infection was more successful than surgery without a toothbrush. In the majority of patients in the Toothbrush group, only one I&D procedure was required. Toothbrushing was associated with a lower rate of surgical failure (implant loosening requiring revision surgery for infection), and infection control was achieved more quickly, as evidenced by faster improvement in infection-related laboratory markers.

This study showed the effectiveness of toothbrush use in 60 patients with SSI after 1081 spinal fusion surgeries. Based on statistical and clinical results, we confirmed that biofilm removal using a toothbrush, in combination with conventional treatment, during early I&D treatment of postoperative spinal infection led to a significantly shorter CRP normalization period and reduced the need for revision surgery, when compared with conventional treatment alone. The effectiveness of toothbrushing was maximized by removing biofilm before implant loosening or bony collapse around the implants. Importantly, we observed no adverse effects related to use of the toothbrushing technique.

Dental plaque is an archetypical biofilm composed of a complex microbial community [43]. According to McCracken et al. [47], plaque can be effectively removed when brushing with a force of 150 g or more for 120 s through toothbrushing. This can be applied in this study, and additional research is needed on the effective brushing time and force for the spine instrument.

Smakman et al. [48] reported that dead bacterial cells can be nutrients for growing other bacteria. When considering electron micrographs and clinical progress, mechanical debridement is possible for brushing. Therefore, biofilm and dead cell debris are also removed. However, if betadine soaking is performed, there is a possibility that dead cells and remnants of biofilm remain and still serve as a medium for bacterial adhesion and growth. Therefore, the risk of recurrence or persistence of infection is high.

This study had some limitations. First, it was difficult to quantify how much biofilm was removed with the toothbrush during surgery and to determine whether the amount of biofilm removal was sufficient. However, it has been reported that biofilm production begins in a few hours after infection, and contact with a foreign surface induces changes in gene expression [49]. Thus, it is likely that all infections already had a biofilm, even in the absence of clinical symptoms. Second, because of the retrospective design of this study, the same number of patients could not be matched for each surgical site, and it was not possible to examine all possible risk factors for spinal infection. Finally, many postoperative infections appear in immunosuppressed people, such as those taking immunosuppressants [50], who were excluded from our study. Thus, additional research is warranted to examine the effectiveness of our toothbrushing technique in these higher-risk patients.

Despite these limitations, we suggest that our toothbrushing procedure is an easy and useful technique for treating patients with SSI after spinal fusion surgery. It may be especially useful for patients who are unable to undergo conventional combined anterior and posterior surgery because of multiple comorbidities, multiple-level infectious lesions, and poor general condition.

## 5. Conclusions

SSIs after spinal fusion can lead to devastating morbidity and mortality. The use of an intraoperative toothbrushing technique during the first I&D surgery of SSIs after spinal fusion surgery can reduce the failure rate and need for revision surgery by effectively removing the biofilm of pathogenic bacteria. We suggest that this easy and convenient method is an excellent option for treating spinal infections.

## Figures and Tables

**Table 1:** Patient characteristics of the study groups.

Characteristics	Total (*n* = 60)	Toothbrush Group (*n* = 20)	No-Toothbrush Group (*n* = 40)	*p*-Value
Age (y)	69.0 (55.0–89.0)	66.5 (58.0–89.0)	71.0 (55.0–82.0)	0.0461 *
Sex				0.0935
Male	36 (60.00)	9 (45.00)	27 (67.50)	
Female	24 (40.00)	11 (55.00)	13 (32.50)	
Body mass index (kg/m^2^)				0.5680
<25	35 (58.33)	15 (75.00)	20 (50.00)	
25–29.9	19 (31.67)	4 (20.00)	15 (37.50)	
≥30	6 (10.00)	1 (5.00)	5 (12.50)	
Smoking				0.4714
Yes	10 (16.67)	5 (25.00)	5 (12.50)	
No	50 (83.33)	15 (75.00)	35 (87.50)	
Hypertension				0.5820
Yes	33 (55.00)	10 (50.00)	23 (57.50)	
No	27 (45.00)	10 (50.00)	17 (42.50)	
Diabetes mellitus				0.7144
Yes	28 (46.67)	10 (50.00)	18 (45.00)	
No	32 (53.33)	10 (50.00)	22 (55.00)	
Renal disease				0.0986
Yes	16 (26.67)	8 (40.00)	8 (20.00)	
No	44 (73.33)	12 (60.00)	32 (80.00)	
Pulmonary disease				>0.9999
Yes	9 (15)	3 (15.00)	6 (15.00)	
No	51(85)	17 (85.00)	34 (85.00)	
Surgical site				0.1750
Cervical	7 (11.67)	2 (10.00)	5 (12.50)	
Thoracolumbar	22 (36.67)	9 (45.00)	13 (32.50)	
Lumbar	20 (33.33)	4 (20.00)	16 (40.00)	
Lumbosacral	11 (18.33)	5 (25.00)	6 (15.00)	
Number of instrumented fusion levels	2.00 (1.00–7.00)	2.00 (1.00–6.00)	2.00 (1.00–7.00)	0.7312
Estimated blood loss during initial fusion surgery (mL)	450 (30–2500)	490 (100–2500)	400 (30–2500)	0.0884
Transfusion (packed RBCs) during initial fusion surgery				0.0792
Yes	14 (23.33)	3 (15.00)	11 (27.50)	
No	46 (76.67)	17 (85.00)	29 (72.50)	
Pre-1st I&D surgery WBC count (×10^3^ cells/µL)	7.12 (2.78–18.62)	6.97 (2.78–18.62)	8.44 (4.24–17.91)	0.2141
Pre-1st I&D surgery ANC (cells/µL)	4217.80 (1278.49–7944.78)	3980.71 (1765.12–7944.78)	4324.96 (1278.49–6823.21)	0.3645

Data are median (minimum–maximum) or number (%). * *p*-value < 0.05. Abbreviations: RBC, red blood cell; WBC, white blood cell; ANC, absolute neutrophil count; I&D, incision and drainage.

**Table 2:** Isolated microbes in surgical site infections.

Microorganisms	Total (*n*)	Toothbrush Group (*n*)	No-Toothbrush Group (*n*)
*Staphylococcus* *epidermidis*	15	4	11
*Staphylococcus aureus*	8	3	5
Methicillin-resistant coagulase-negative *Staphylococcus*	4	2	2
Methicillin-resistant *Staphylococcus aureus*	9	2	7
*Pseudomonas aeruginosa*	3	2	1
*Klebsiella pneumonia*	3	-	3
*Escherichia coli*	5	2	3
*Enterococcus faecalis*	4	2	2
*Candida parapsilosis*	1	1	-
*Mycobacterium* *tuberculosis*	-	-	-
No growth	8	2	6

**Table 3:** Clinical outcomes of the study groups.

Clinical Outcomes	Total (*n* = 60)	Toothbrush Group (*n* = 20)	No-Toothbrush Group (*n* = 40)	*p*-Value
Postoperative hemovac removal after I&D surgery (POD)	7.0 (5.0–14.0)	7.0 (5.0–12.0)	7.0 (5.0–14.0)	0.3742
Cumulative hemovac volume after I&D surgery (mL)	410 (20–2600)	440 (20–2600)	350 (80–1400)	0.1160
Post I&D surgery serum glucose (mg/dL)	130.8 (57–284)	146.7 (92–284)	112.8 (57–198)	0.7522
Post I&D surgery serum albumin (g/dL)	2.9 (1.80–4.94)	3.3 (1.80–4.94)	2.8 (2.20–4.54)	0.2021
Time from fusion surgery to diagnosis of SSI (days)	75.0 (10.0–500.0)	45.0 (10.0–500.0)	90.0 (12.0–450.0)	0.0796
Early SSI (<30 days)	34 (55.00)	12 (60.00)	22 (55.00)	
Late SSI (≥30 days)	26 (45.00)	8 (40.00)	18 (45.00)	
Time from SSI diagnosis to 1st I&D surgery (days)	8.0 (1.0–90.0)	10.0 (2.0–30.0)	7.5 (1.0–90.0)	0.4074
Total time to CRP normalization after 1st I &D surgery (days)	24.0 (10.0–90.0)	20.0 (10.0–50.0)	27.5 (14.0–90.0)	0.0449 *
Duration of intravenous antibiotics usage in the hospital perioperative 1st I&D surgery (days)	25.0 (10.0–90.0)	20.0 (12.0–58.0)	26.0 (10.0–90.0)	0.2082
Duration of oral antibiotics usage after hospital discharge (days)	15.75 (7.0–28.0)	16.45 (7.0–28.0)	15.4 (7.0–28.0)	0.4871
Need for revision surgery after 1st I&D surgery				0.0395 *
Yes	16 (26.67)	2 (10.00)	14 (35.00)	
No	44 (73.33)	18 (90.00)	26 (65.00)	

Data are median (minimum–maximum) or number (%). * *p*-value < 0.05. Abbreviations: RBC, red blood cell; WBC, white blood cell; ANC, absolute neutrophil count; I&D, incision and drainage.

## Data Availability

All data are reported in the manuscript.
